# Impact of Socio-economic Status on Low Birthweight: Decomposing the Differences Between Natives and Immigrants in Spain

**DOI:** 10.1007/s10903-020-01027-0

**Published:** 2020-05-14

**Authors:** Mikolaj Stanek, Miguel Requena, Alberto del Rey

**Affiliations:** 1 Department of Sociology and Communication, University of Salamanca, Edificio FES, Campus Miguel de Unamuno, 37007 Salamanca, Spain; 2 Department of Social Structure, UNED, Madrid, Spain

**Keywords:** Birthweight, Spain, Healthy immigrant, Socio-economic status

## Abstract

In this population-based study, we explored the relationships between immigration, socio-economic status (SES), and perinatal outcomes. We quantified the effects of SES on birthweight disparities between native and immigrant mothers in Spain. We obtained birth and SES data from the 2011 census and administrative registers for years 2011–2015. The associations between origin, statuses, and the likelihood of low birthweight were estimated using logistic regressions. Fairlie’s nonlinear extension of the Oaxaca–Blinder decomposition method was applied to identify the extent to which the differences in birthweight between groups corresponded to socio-economic composition or to rates. Our results showed that African and Latin American mothers exhibited advantage in the perinatal outcomes over native mothers [odds ratio (OR) 0.75; 95% confidence interval (CI) 0.63–0.90 and OR 0.73; 95% CI 0.65–0.82, respectively]. Decomposition analyses revealed that such advantage was not affected by the lower positions within the socio-economic structure that African and Latin American populations occupied.

## Background

Socio-economic status (SES) is known to strongly affect health outcomes [1, 2]. In particular, the link between SES and perinatal health is well established. A low SES is typically associated with poor economic conditions, physically demanding occupations, increased likelihood of unhealthy behaviors and exposure to chronic stressors, and decreased utilization of healthcare services [3, 4], all of which are considered as risk factors for undesirable birth outcomes. Mothers of immigrant background are more likely to occupy lower positions in the socio-economic structure (low SES) compared with native mothers [5, 6] and exhibit an increased risk of adverse health outcomes. Nevertheless, a considerable amount of research show that some categories of immigrant women benefit from better pregnancy outcomes than native women despite their lower socioeconomic status [7–10]. More specifically, some research showed that, some immigrant groups are more resistant to negative birth outcomes that are typically associated with low educational or occupational attainments [9, 11]. Thus, the socio-economic gradient may not predict the perinatal outcomes in the immigrant populations in the same way as in the native population. This regularity is referred to in the literature as the healthy immigrant effect [12, 13]

Although the healthy immigrant effect depends strongly on specific outcomes and the origins of the immigrants, a number of studies showed that immigrant status played a protective role in gropus which are particularly exposed to adverse socio-economic penalties. Specifically, some immigrant groups exhibited more favorable perinatal health outcomes when compared to natives and other immigrant populations even if they are more socioeconomically vulnerable [14–16]. In addition, there is evidence that the protective effect of immigrant status regarding specific indicators of perinatal healts variates across SES categories within the same immigrant groups. For example, Acevedo-Garcia et al. [11] showed that foreign born status was associated with high odds of low birth weight among highly educated mothers and lower odds among mothers with low educational attainment. Similar patterns were observed for specific origins in Belgium [16].

In the last two decades Spain has experienced a remarkable increase in immigration flows. As a result, immigrant population residing in Spain has grown from 1.6% in 1998 to 14.3% in 2019. Massive arrivals of immigrants have raised interest in their contribution to fertility levels of the Spanish population and, consequently, in several issues related to the reproductive health of immigrant mothers [17–19]. However, most of the studies on perinatal outcomes among immigrant populations in Spain have focused on exploring the healthy migrant effect by comparing birth outcomes between foreign-born and Spanish women [20–24]. In comparison, less attention has been paid to the impact of SES [25, 26].

The objective of this study was to quantitatively assess the contribution of socio-economic disparities to the differences in perinatal outcomes between native and immigrant mothers in Spain. We analyzed the observable socio-economic disparities to assess in which immigrant groups and to which extent being an immigrant protected against the negative impact of a low SES on perinatal health. Our analysis is guided by three assumptions based on previously cited research. First, we expect that the perinatal outcoms will vary according to mother’s origin. Second, low SES negatively affects birth outcomes. Finally, we expect that disparities in SES composition origins will account for a significant part of the differences in perinatal health between Spanish and immigrants.

## Data and Methods

### Participants

We used a new dataset extracted from the births administrative registers and the 2011 Spanish census. This dataset, available upon request at the Spanish National Statistical Office (Instituto Nacional de Estadística), links individual birth information between 2011 and 2015 (Movimiento Natural de la Población) to data of the 2011 census, which include the personal and household characteristics of the individual such as sex, age, country of origin, marital status, education, labor market situation, migratory status and living conditions. This dataset includes approximately 10% of the Spanish population living in households based on the 2011 census and excludes births from mothers who are not recorded in the census (including those living in institutions or abroad). For our analytical purposes, we selected living singleton babies born to Spanish and immigrant mothers. We excluded outliers in terms of weights and gestational age, such as macrosomia and preterm births. Consequently, 128,720 births were included in this analysis (Table 1). This study did not require ethical approval (anonymized national data).

**Table 1:** Descriptive statistics by mother’s origin

	Spain	EU15 and other developed	Other European	African	Latin American
*Number of births*	115,842	2202	2085	2903	6765
*With low weight*	6634	133	126	148	283
*Sex of child (%)*					
Male	51.62	50.82	52.09	52.39	52.74
Female	48.38	49.18	47.91	47.61	47.26
*Birth order (%)*					
1	87.99	87.74	92.13	82.05	92.13
2	11.66	11.67	7.53	15.16	7.66
≥ 3	0.34	0.59	0.34	2.79	0.21
*Mothers’ age at birth (%)*					
< 25	5.14	3.41	13	16.4	14.27
25–34	52.37	44.32	65.42	55.84	50.79
≥ 35	42.5	52.27	21.58	27.76	34.94
*Mothers' partnership status at birth (%)*					
Married	65.01	59.81	63.36	86.5	54.37
Consensual union	15.4	18.57	18.71	2.24	19.97
Never married	19.59	21.62	17.94	11.26	25.66
*Mothers' education 2011 (%)*					
Primary or less	26.05	18.39	35.68	74.27	34.66
Secondary+	73.95	81.61	64.32	25.73	65.34
*Maternal labour market status 2011 (%)*					
Occupied	67.82	65.76	42.01	16.12	45.95
Unemployed	24.11	23.66	39.95	43.02	35.07
Inactive	8.07	10.58	18.03	40.85	18.98
*Highest occupational status in household 2011%*					
High	33.91	40.01	9.11	4.2	17.39
Medium	38.24	37.6	29.54	16.47	30.14
Low	27.85	22.39	61.34	79.33	52.46

### Measures

Outcome: We used birthweight (in grams) as our main outcome measure. We categorized birthweight into two groups: low birthweight (LBW: < 2500 g) and normal weight (2500–3999 g).

Exposures: We classified maternal origin into four groups according to the geographical proximity and basic economic indicators: (1) Spanish (2) EU15 and other highly developed countries which include EU member states prior to the 2004 enlargement, Norway, Iceland, Switzerland, Canada, United States, Australia, New Zealand, Japan, South Korea, Taiwan, and Singapore; (2) other European countries; (3) African countries; and (4) Latin American countries. We included three SES measures (provided by the 2011 Census), namely maternal education (less than secondary and at least secondary), maternal employment status (employed, unemployed, and inactive), and the highest occupational category among household members (low, medium, and high). In addition, we included other covariates related to socio-demographic features provided by the birth register for each year, namely the sex of the new-born, birth order, maternal age at birth, and mothers’ partnership status at birth.

### Analysis

We used two analytical approaches. First, we applied logistic regression models to estimate the effects of the different exposures and, in particular, of the country of origin and the socio-demographic and socio-economic variables on the likelihood of delivering an LBW baby. Second, using a regression-based decomposition technique for non-lineal models [27], we performed a decomposition of differences in LBW observed between natives and several immigrant groups. This technique allows the differences to be decomposed into two portions: (1) the “composition effect” or “explained component” that corresponds to the socio-demographic and socio-economic compositions; and (2) the “coefficient effect” or “unexplained component” that corresponds to the other factors associated with LBW prevalence that are specific to these groups. The explained component accounts for the part of the difference that is attributable to the structural dissimilarities between the groups, whereas the unexplained component accounts for the behavioral differences between the groups that are not attributable to the compositional factors. In other words, the composition component indicates the differences in LBW between the groups if the coefficients (rates) of LBW in each group had been the same while only the socio-economic characteristics varied across groups. The coefficient effect (or the unexplained component) estimates the difference that cannot be explained by the dissimilarities in the group compositions (which may be due to other specific behaviors in the groups).

## Results

Table 1 summarizes the specific features of the four immigrant categories. Compared with native mothers, LBW prevalence was slightly higher in immigrants from EU15 and other highly developed countries and from less developed European countries and lower in immigrants from Africa and Latin America. We observed considerable variations in specific socio-economic features between the groups. Mothers from developed countries had noticeably higher levels of educational attainment (81.6% with secondary or tertiary education) compared with the natives (74.0%) and other immigrant categories. Mothers of African background had by far the lowest levels of educational attainment (only 25.7% with secondary or tertiary education). Approximately two thirds of mothers born in Spain and in highly developed countries were employed, and slightly less than a quarter were unemployed. Almost 46% of Latin American mothers and 42% of mothers from other European countries were employed, and 35% and nearly 40% were unemployed, respectively. African-born mothers had the highest rates of unemployment and inactivity. The households of mothers from highly developed countries exhibited the highest levels of occupational status, followed by the households of Spanish and Latin American mothers. The household occupational status of African mothers exhibited the most uneven distribution with almost 80% having a low SES.

**Fig. 1:**
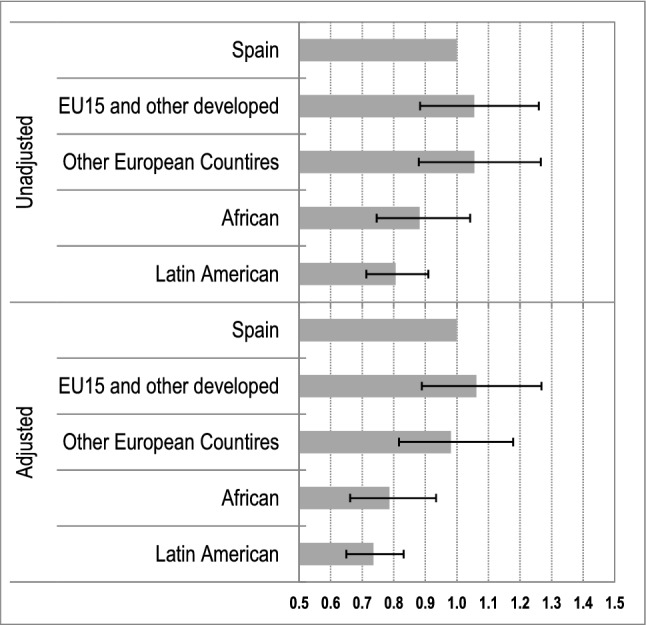
ORs of LBW by the country of origin (horizontal bars) with 95% CI (horizontal lines). Unadjusted and adjusted ORs by the sex of the child, birth order, maternal age, maternal marital status, maternal educational attainment, maternal labor market status in 2011, and the highest household occupational status in 2011

Figure 1 shows the adjusted and unadjusted odds ratios (ORs) of LBW estimated using logistic regression models with the country of origin as the main exposure. The adjusted ORs showed that African and Latin American mothers were less likely to deliver LBW children compared with Spanish mothers. When controlling for several socio-demographic and socio-economic covariates, African and Latin American mothers were 21% [OR 0.79; 95% confidence interval (CI) 0.66–0.93] and 26% (OR 0.74; 95% CI 0.65–0.83] less likely to deliver LBW infants, respectively. We observed no significant differences in LBW deliveries between mothers born in Spain and mothers immigrating from from EU15 and other highly developed countries and from other European countries (see Fig. 1).

Table 2 shows the logistic regression models with detailed ORs for the socio-economic and socio-demographic variables. As expected, we observed more LBW in female babies, second and third birth orders, women aged less than 25 years, and married mothers. The effects of SES indicators were in line with previous findings regarding social gradient and the risk of infant LBW. Compared with mothers with low educational attainment, mothers with a high educational attainment were 19% less likely to deliver LBW babies. Compared with a low household occupational status, a high and medium household occupational status also reduced the likelihood of LBW by 22% and 7.5%, respectively. Moreover, the probability of LBW was 11% higher in unemployed mothers than that in employed mothers. These tendencies were also observed in African and Latin American mothers, with mothers of low SES more likely to deliver LBW babies.

**Table 2:** Logistic regression models predicting low birthweight: adjusted odds ratios

	All			Spain			African			Latin American		
	OR	95% CI		OR	95% CI		OR	95% CI		OR	95% CI	
Origin												
Spain (ref.)												
EU15 and other developed	1.047	0.877	1.251									
Other European	0.971	0.809	1.167									
African	0.755	0.633	0.900*									
Latin American	0.730	0.645	0.825***									
*New-born sex*												
Male (ref.)												
Female	1.236	1.179	1.296***	1.229	1.170	1.292***	1.577	1.126	2.210**	1.175	0.925	1.493
*Birth order*												
1 (ref.)												
2	0.861	0.795	0.933***	0.873	0.803	0.948**	0.810	0.487	1.346	0.733	0.437	1.227
≥ 3	0.877	0.585	1.313	0.843	0.531	1.338	0.955	0.340	2.679	1.490	0.191	11.639
*Maternal age at birth*												
< 25 (ref.)												
25–34	0.895	0.809	0.990*	0.876	0.784	0.978*	1.408	0.810	2.447	0.745	0.515	1.079
≥ 35	1.040	0.937	1.154	1.006	0.898	1.127	2.071	1.165	3.681*	0.997	0.677	1.467
*Maternal family status at birth*												
Married (ref.)												
Consensual union	1.242	1.164	1.325***	1.261	1.178	1.350***	2.610	1.205	5.652*	1.097	0.795	1.512
Never married	1.193	1.123	1.268***	1.203	1.129	1.283***	0.839	0.476	1.480	1.126	0.834	1.521
*Maternal education 2011*												
Primary (ref.)												
Secondary+	0.807	0.738	0.881***	0.831	0.753	0.917***	0.664	0.469	0.939**	0.678	0.485	0.948
*Maternal labour market status 2011*												
Occupied (ref.)												
Unemployed	1.113	1.051	1.178***	1.120	1.055	1.190***	1.003	0.619	1.626	0.980	0.743	1.294
Inactive	1.038	0.952	1.132	1.048	0.953	1.152	0.920	0.559	1.516	1.039	0.741	1.457
*Highest occupational status in household 2011*												
Low (ref.)												
Medium	0.925	0.872	0.981**	0.931	0.876	0.990*	0.924	0.584	1.461	0.841	0.629	1.124
High	0.784	0.734	0.838**	0.771	0.719	0.826***	0.615	0.219	1.731	1.160	0.832	1.616
*Constant*	0.071	0.062	0.081***	0.071	0.061	0.081***	0.036	0.017	0.073***	0.072	0.043	0.121***
*Number of observations*	128,720			115,482			2903			6048		
*Log likelihood*	− 27919.975			− 25223.747			− 570.105			− 1132.614		

Table 3 summarizes the decomposition of the differences in LBW prevalence between pairs of origin groups. We decomposed the differences in LBW between Spanish and African as well as between Spanish and Latin Americans mothers. The upper panel reports the rates of LBW among origin categories and the differences between them. It also shows how much of this difference was attributable to the differences in observable characteristics between the groups (explained portion) and how much remains unexplained (coefficient effects). The lower panel shows the contribution to the differences in the compositional factors corresponding to observable characteristics and, specifically, the relative contribution of each variable included in the model to the overall differences.

**Table 3:** Non-linear decomposition of the differences in low birthweight between Spanish and immigrant mothers

	Spain vs African			Spain vs Latin American		
	Coef	[95% CI]		Coef	[95% CI]	
Spanish mother	0.0574	0.0561	0.0588	0.0574	0.0561	0.0588
Immigrant mother	0.0510	0.0430	0.0590	0.0468	0.0415	0.0521
Difference	0.0065	− 0.0016	0.0146	0.0107	0.0052	0.0161
Explained portion	− 0.0058	− 0.0082	− 0.0033	− 0.0046	− 0.0056	− 0.0036
Unexplained portion	0.0122	0.0044	0.0201	0.0153	0.0098	0.0207
Contribution of each variable to explained portion						
Sex of child	0.0001	− 0.0001	0.0003	0.0001	0.0000	0.0003
Birth order	0.0005	− 0.0001	0.0010	− 0.0003	− 0.0005	− 0.0001
Maternal age at birth	0.0006	− 0.0001	0.0013	0.0000	− 0.0005	0.0006
Maternal family status at birth	0.0025	0.0019	0.0030	− 0.0011	− 0.0015	− 0.0008
Maternal education 2011	− 0.0045	− 0.0060	− 0.0029	− 0.0009	− 0.0012	− 0.0006
Maternal employment status 2011	− 0.0017	− 0.0036	0.0002	− 0.0008	− 0.0016	− 0.0001
Highest occupational category in household 2011	− 0.0032	− 0.0045	− 0.0018	− 0.0016	− 0.0022	− 0.0009

The results of the decomposition of the differences in LBW prevalence between Spanish and African mothers showed that the gap in birthweight (0.0065), which was composed of positive and negative terms, was mainly driven by the unexplained components. The negative value (− 0.0058) obtained for the explained portion suggested that the observable characteristics reduced this disadvantage for Spanish mothers in comparison with that for African mothers. This typically happens when the more disadvantaged group (in this case, Spanish mothers) has some advantage in some observed characteristics (higher SES). A more detailed decomposition (lower panel) revealed that the higher SES and educational levels in Spanish mothers were responsible for the reduction in this disadvantage. The analysis of the LBW gap (0.0107) between Spanish and Latin American mothers revealed a similar pattern. The estimated explained portion of the difference was negative (− 0.0046), suggesting that Spanish mothers had an advantage in their observable characteristics over Latin American mothers. Likewise, the disparities in SES (maternal educational attainment and occupational category in particular) accounted for the main part of the explained portion of the differences.

The values for the unexplained portion of the differences between Spanish and African mothers as well as Spanish and Latin American mothers were positive (+ 0.0122 and + 0.0153 respectively), suggesting that the lower LBW prevalence in African and Latin American populations was relatively independent of their socio-economic distributions (Table 2).

## Discussion

Our study provided three main findings. First, consistent with previous findings in Spain and other countries, our study confirmed that the patterns of perinatal outcomes were highly dependent on the origin of the groups and strongly outcome-specific [9, 21, 22, 24, 28]. Regarding birthweight, mothers from EU 15 and highly developed countries and other European countries had similar outcomes in infant LBW prevalence. However, LBW was less prevalent among African and Latin American mothers. Second, in line with other studies [3], our logistics regression models showed that a low SES negatively affected birth outcomes. However, these effects varied based on specific dimensions of the SES. Education seemed to be the most important factor for predicting poor perinatal outcomes. Indeed, a low educational attainment typically correlates with increased unhealthy behaviors and decreased utilization of healthcare services [29]. Third, the compositional differences associated with the SES contributed to the observed disparities in LBW between native and immigrant mothers. Ceteris paribus, a higher socio-economic attainment of Spanish mothers likely reduced their disadvantage in LBW in comparison with African and Latin American mothers. In other words, if Spanish mothers were given the socio-economic characteristics of African and Latin American mothers, the differences in LBW between the natives and the two immigrant groups would have been greater. Interestingly, this advantage observed in Latin American and African mothers was strongly driven by unexplained factors and not by the SES.

Although it was not possible to clearly identify the protective factors that counteracted the negative impact of the SES, our results provided some insights regarding the possible accounts of the healthy immigrant effect. One possibility is the operation of a health selection effect: immigrants do not fully represent the population of their country of origin. People who choose to emigrate are typically healthier and have a higher SES than people who do not [12, 25]. However, a direct comparison between emigrant mothers and their fellow countrywomen in African and Latin American countries is required to confirm this hypothesis. Another potential complementary explanation to our findings is that certain immigrant groups may exhibit specific socio-cultural traits (traditions, behaviors, and norms) that make positive perinatal outcomes more probable [30]. Several studies have suggested that some migrant groups exhibit protective health behaviors based on internal social norms and social ties that may reduce LBW even under adverse socio-economic conditions, including healthier diets, lower consumption of cigarettes and alcohol, extended living arrangments and better social support from family members and their ethnic communities [31]. Future research should consider such contextual social data regarding the intersection between health behaviors and social networks’ dynamics within different immigrant groups.

### Contribution

Our analysis provided new insights into the sources of perinatal health disparities by separating the potentially adverse impact of socio-economic heterogeneity from other factors. The most frequently used SES indicator is educational attainment. However, SES is a complex and multidimensional phenomenon that also involves income levels and occupation [2]. Each of these dimensions can provide different resources and displays different relationships with health outcomes [29]. Indeed, unidimensional SES measurements may not comprehensively reflect the stratification in the access to vital resources (which may also affect birth outcomes) [9]. To overcome this limitation, we adopted a multidimensional SES analysis approach and analyzed three different SES measures. Our results showed that although the educational attainment was the best SES predictor of birth outcomes, the other two measures also played significant roles and therefore should be included in future research. Moreover, by applying the decomposition based on Fairlie’s technique, which is rarely used to explain the differences in birth outcomes in an ethnically diverse context [32], we overcame the usual limitations of using only regression models and quantified the amount and direction of contributions of the unobserved factors.

### Limitations

Our study has some limitations. First, the birth register lacks potential relevant data regarding the health behaviors of the mothers (such as smoking), utilization of the healthcare systems, and health conditions (such as obesity and diabetes). Second, the limited sample size in our study (10% of the 2011 Census and birth data) did not allow us to analyze the full extent of the origin heterogeneity in Spain (in terms of individual countries). To overcome this limitation, we clustered the origins of immigrants into larger categories based on the geographical and economic status. Third, as our study was based on the birth data for the period 2011–2015 and several socio-economic indicators provided by the 2011 Census, changes in these indicators over time were not considered.

## Conclusions

This study provides new evidence on the complex relationship between the SES and LBW prevalence between natives and immigrants in Spain. First, certain immigrant populations (e.g., Africans and Latin Americans) but not others (e.g., non-EU15 Europeans) have a perinatal advantage over the natives. Second, although there is a negative association between birth outcomes and the SES, the observed perinatal advantage in the African and Latin American populations over the native Spanish population is not affected by their positions in the socio-economic structure. These findings are compatible with several explanations for the immigrant perinatal health paradox. The fact that migrants are favourably selected in origin does not exclude the possibility that other socio-cultural features and behaviors may contribute to these perinatal advantages. Further research is needed, including when possible the consideration of genetic factors, to clarify the healthy immigrant paradox.
